# Beyond the Image—Advancing Culturally Safe Radiology for Aboriginal and Torres Strait Islander Health

**DOI:** 10.1111/1754-9485.13871

**Published:** 2025-06-04

**Authors:** Shabnam Mohamad Shafiq, Amlan Chowdhury, Ji Woo Kim

**Affiliations:** ^1^ University of Notre Dame Australia School of Medicine Darlinghurst, Sydney Australia; ^2^ University of Auckland Medical School Auckland New Zealand

**Keywords:** applied imaging technology, clinical and cost benefit, intervention, radiation oncology imaging, radiography

## Abstract

Despite the growing recognition of cultural safety in Australian healthcare, radiology remains an area where Aboriginal and Torres Strait Islander peoples continue to face substantial barriers. Although medical education has integrated broader Indigenous health training, clinical radiology has often been overlooked. The common misconception that radiology is a technical, patient‐detached specialty has contributed to a lack of culturally competent training for radiologists and radiographers. Given the high burden of diseases requiring imaging, such as chronic respiratory illness, otitis media, cardiovascular disease and trauma‐related injuries, culturally safe radiological services are vital for achieving health equity.

## 1 The State of Current Research in Australia

The need for cultural safety training in healthcare is well established, yet radiology‐specific efforts remain limited. Research demonstrates that Aboriginal and Torres Strait Islander patients frequently experience delayed diagnoses due to factors, such as geographic isolation, mistrust of healthcare institutions and financial hardship [1, 2]. Many report feeling culturally unsafe in medical environments, which further discourages engagement with diagnostic services [3]. Despite this, few studies have explored how radiology training programmes prepare clinicians to address these challenges.

Medical schools in Australia often focus Indigenous health content on primary care and public health, with limited content directed at specialties like radiology [4]. Where efforts exist, they are inconsistent across institutions. A more detailed investigation into radiology professionals' attitudes towards cultural competency and strategies for integrating it effectively into curricula is needed.

## 2 Real‐World Barriers and Their Impact on Patient Outcomes

### 2.1 Geographic and Logistical Barriers

Many Aboriginal and Torres Strait Islander communities live in remote regions where access to imaging is scarce. Patients may need to travel hundreds of kilometres to reach services, contributing to diagnostic delays and worsened outcomes [5]. Although mobile imaging units exist, their sporadic availability often fails to meet local demand.

A case study from Far North Queensland revealed that radiographers working solo in remote hospitals had to be culturally competent, use local dialects and adapt their scope of practice to improve care quality and accessibility for Indigenous patients [6]. Similarly, the ‘better access to medical imaging’ initiative in Torres and Cape regions brought ultrasound and mobile x‐ray services to clinics that had never previously offered imaging. Patient feedback was overwhelmingly positive, citing the elimination of long‐distance travel and increased comfort in receiving care within their communities [7, 8].

### 2.2 Cultural Safety Concerns

Imaging procedures often involve close physical proximity, unfamiliar machines, and language barriers, which may be distressing without adequate support. In the absence of culturally trained staff, this can lead to incomplete scans or avoidance of services altogether [6]. Historical injustices in medical care contribute to deep mistrust and reluctance to access mainstream services.

Direct patient testimonies reinforce these concerns. A Yolngu woman from Arnhem Land described feeling ‘treated like a number’ during an imaging appointment, stating that ‘no one explained what was going on, and they did not let me ask questions’. Likewise, Aboriginal cancer patients have expressed that emotional support and culturally safe communication had a greater impact than clinical treatments alone. One patient remarked, ‘Yeah, the support has been brilliant… that helps me more than the chemo’ [9]. Another highlighted the critical role of Indigenous Liaison Officers in making them feel safe and understood, stating, ‘They have done all they can to keep me here’ [9].

## 3 Practical Solutions and the Role of Medical Education

### 3.1 Embedding Indigenous Led Training in Radiology Curricula

Radiology programmes must incorporate Indigenous‐led, case‐based teaching that centres Aboriginal and Torres Strait Islander perspectives. Indigenous health professionals and community leaders should design and deliver this content to ensure authenticity. Similar approaches have shown success in general practice and emergency medicine [10]. Existing Indigenous radiology staff can also serve as internal champions for such initiatives, helping to embed cultural safety as standard practice.

### 3.2 Expanding Mobile and Community‐Based Imaging Services  

Mobile imaging services should be expanded and integrated into ongoing outreach programmes. The Royal Flying Doctor Service and Deadly Choices have shown that outreach healthcare improves access and trust. Investment must ensure radiology services are included in these models with consistent schedules, appropriate staffing and culturally safe settings [7, 8].

### 3.3 Increasing the Representation of Indigenous Radiology Professionals

Workforce diversity enhances patient care. Recruitment programmes, mentorships and scholarships should target Aboriginal and Torres Strait Islander students for careers in radiology. These professionals bring lived experience and community connection that enrich the cultural safety of the profession.

### 3.4 Enhancing Interdisciplinary Collaboration

Collaboration between radiologists and general practitioners, Aboriginal Health Workers, nurses and allied health staff ensures a holistic understanding of patients' cultural needs. Integrating cultural safety into team‐based care can create a more inclusive and respectful environment across the diagnostic pathway.

### 3.5 Improving Communication Skills and Cultural Responsiveness

Effective communication is foundational to culturally safe care. Radiology staff must be trained in working with interpreters, understanding the importance of family and gender considerations, and recognising different conceptions of health and illness [11, 12].

## 4 Recommendations for Future Research

While the article calls for more research, specific frameworks can guide this effort:

- *Methodologies*: Participatory Action Research and Community‐Based Participatory Research are ideal for working in partnership with Indigenous communities.
- *Settings*: Research should span metropolitan, regional and remote contexts, and include both public and private healthcare settings.
- *Funding*: Dedicated grants from the National Health and Medical Research Council (NHMRC) and Indigenous‐led research funding bodies should be prioritised.
- *Collaboration*: Projects must involve Aboriginal and Torres Strait Islander communities from conception through to dissemination to ensure relevance and impact.

## 5 Policy Implications

Systemic change requires policy reform at institutional and governmental levels. Accreditation bodies, such as the Royal Australian and New Zealand College of Radiologists (RANZCR), should mandate cultural safety components in continuing professional development. Health departments must also invest in workforce diversity and equitable service delivery. Embedding these principles into policy frameworks ensures sustainability beyond individual projects or educators.

## 6 Conclusion

Cultural safety in radiology is not a peripheral issue; it is central to equity in healthcare for Aboriginal and Torres Strait Islander peoples. Overcoming current deficits requires a multifaceted approach: reforming medical education, expanding access to services, investing in workforce diversity, and embedding cultural safety at both clinical and policy levels.

By incorporating Indigenous voices, lived experiences and practical solutions, radiology can evolve into a more inclusive and patient‐centred field. Prioritising research, interdisciplinary collaboration and community leadership will help close the healthcare gap and improve outcomes for Indigenous patients across Australia.

## Conflicts of Interest

The authors declare no conflicts of interest.

## Data Availability

Data sharing not applicable to this article as no datasets were generated or analysed during the current study.
